# Food Insecurity, Health, and Development in Children Under Age Four Years

**DOI:** 10.1542/peds.2019-0824

**Published:** 2019-09-09

**Authors:** Chloe R. Drennen, Sharon M. Coleman, Stephanie Ettinger de Cuba, Deborah A. Frank, Mariana Chilton, John T. Cook, Diana B. Cutts, Timothy Heeren, Patrick H. Casey, Maureen M. Black

**Affiliations:** a School of Medicine, University of Maryland, Baltimore, Maryland;; b Biostatistics and Epidemiology Data Analytics Center and Department of Biostatistics, School of Public Health, Boston University, Boston, Massachusetts;; c Department of Pediatrics, School of Medicine, Boston University, Boston, Massachusetts;; d Department of Health Management and Policy, Dornsife School of Public Health, Drexel University, Philadelphia, Pennsylvania;; e Hennepin County Medical Center, Minneapolis, Minnesota;; f Department of Pediatrics, College of Medicine, University of Arkansas for Medical Sciences, Little Rock, Arkansas;; g Department of Pediatrics, School of Medicine, University of Maryland, Baltimore, Maryland;; h RTI International, Research Triangle Park, North Carolina

## Abstract

**BACKGROUND AND OBJECTIVES:**

Food insecurity and pediatric obesity affect young children. We examine how food insecurity relates to obesity, underweight, stunting, health, and development among children <4 years of age.

**METHODS:**

Caregivers of young children participated in a cross-sectional survey at medical centers in 5 US cities. Inclusion criteria were age of <48 months. Exclusion criteria were severely ill or injured and private health insurance. The Household Food Security Survey Module defined 3 exposure groups: food secure, household food insecure and child food secure, and household food insecure and child food insecure. Dependent measures were obesity (weight-age >90th percentile), underweight (weight-age <5th percentile), stunting (height/length-age <5th percentile), and caregiver-reported child health and developmental risk. Multivariable logistic regression analyses, adjusted for demographic confounders, maternal BMI, and food assistance program participation examined relations between exposure groups and dependent variables, with age-stratification: 0 to 12, 13 to 24, 25 to 36, and 37 to 48 months of age.

**RESULTS:**

Within this multiethnic sample (*N* = 28 184 children, 50% non-Hispanic African American, 34% Hispanic, 14% non-Hispanic white), 27% were household food insecure. With 1 exception at 25 to 36 months, neither household nor child food insecurity were associated with obesity, underweight, or stunting, but both were associated with increased odds of fair or poor health and developmental risk at multiple ages.

**CONCLUSIONS:**

Among children <4 years of age, food insecurity is associated with fair or poor health and developmental risk, not with anthropometry. Findings support American Academy of Pediatrics recommendations for food insecurity screening and referrals to help families cope with economic hardships and associated stressors.

Food insecurity, defined as “limited or uncertain availability of nutritionally adequate and safe foods or limited or uncertain ability to acquire acceptable foods in socially acceptable ways,”^1^ is a national public health problem. The US Department of Agriculture estimates that 16.4% of households with children<6 years of age experienced food insecurity in 2017, with higher rates among households headed by single, African American, or Hispanic parents.^1,2^ Food insecurity among young children is associated with poor overall health, hospitalizations, developmental risk, and behavioral problems.^2–6^ Associations between food insecurity and young children’s weight status have been inconsistent, with reports of underweight, overweight, and no relation with weight.^7,8^

Infancy, toddlerhood, and early preschool (0–4 years) represent a period of rapid growth and brain development. Variability in children’s early nutritional status can have lifelong consequences extending into the subsequent generation.^9^ Stunting (length/height-for-age>2 SDs below the median) increases the risk for low school achievement, cognitive deficits, and chronic disease in adulthood.^10–12^ Overweight increases the risk for obesity and associated comorbidities throughout childhood and adulthood.^13^ National data reveal a 20% increase in obesity among kindergarteners from 1998 to 2010, with children of low socioeconomic status at highest risk.^14^

Adequate nutrition, crucial to support healthy growth and development during early childhood, could be threatened by food insecurity.^15^ Previous researchers suggest a nonlinear relationship between food insecurity and weight status among adults: moderate food insecurity leads to reductions in food quality that may result in overweight,^16^ whereas severe food insecurity leads to reductions in food quality and quantity that could result in overweight or underweight in varying contexts.^17–19^

Research addressing food insecurity among children<4 years of age has not been stratified by age, potentially masking important developmental differences in young children’s experience of food insecurity and susceptibility to growth alterations. Young children’s diets, meal patterns, and response to household stress differ significantly by age, suggesting that the experience of food insecurity may vary across developmental stages.^20^ Most research among families of young children has been focused on household food insecurity rather than child food insecurity, which is a more severe form of food insecurity that directly impacts availability of food to children. Efforts aimed at addressing how household and child food insecurity are associated with young children’s growth and development may inform the prevention of food insecurity and both underweight and obesity.^21,22^

This study uses a large sample of children in low-income families stratified by ages 0 to 12, 13 to 24, 25 to 36, and 37 to 48 months to assess age-specific relations between household and child food insecurity and children’s growth, health, and development. We hypothesize that household and child food insecurity are associated with increased age-specific odds of obesity, underweight, stunting, fair or poor health, and developmental risk.

## METHODS

### Sample

**FIGURE 1:**
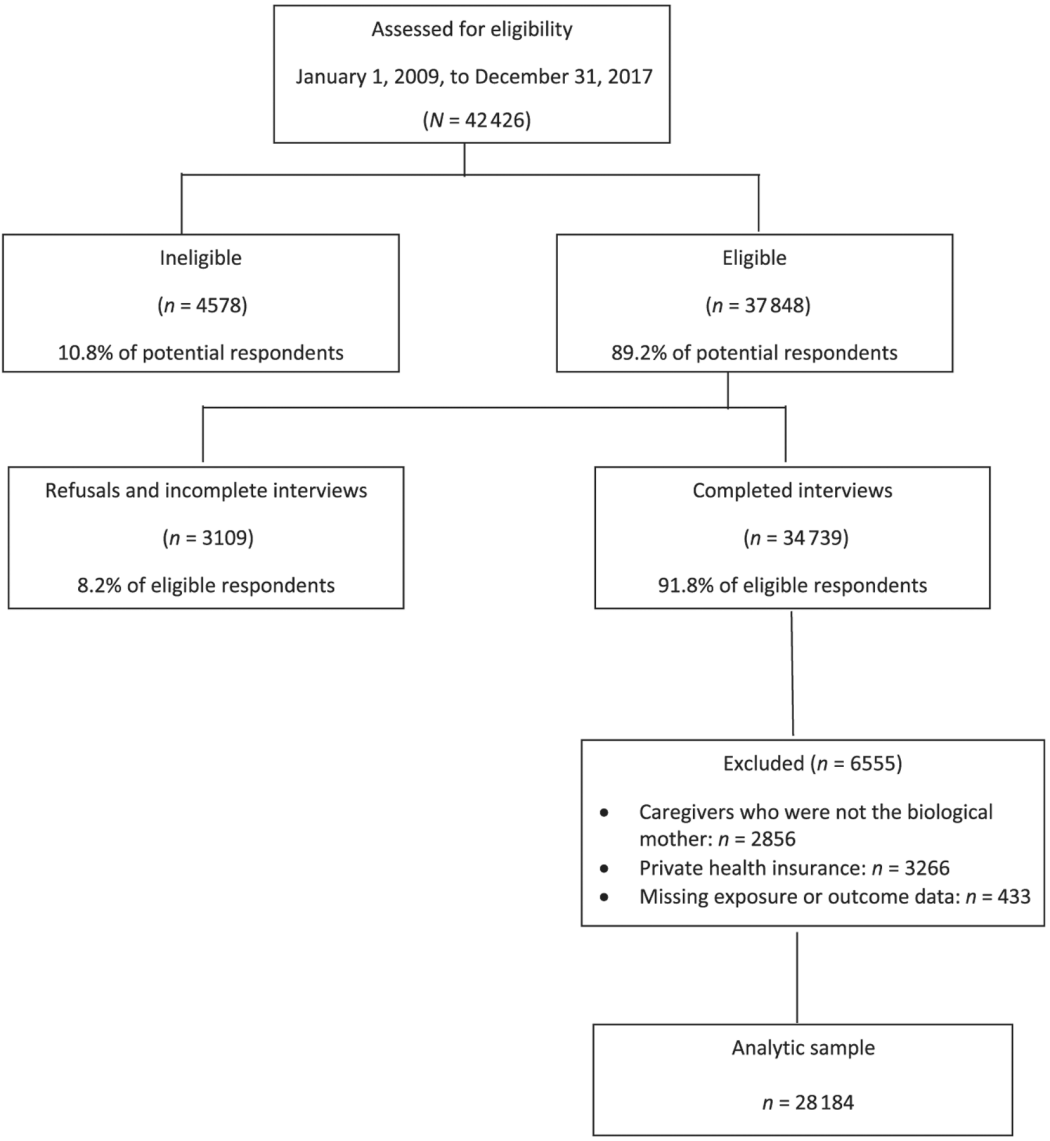
Description of the analytic sample.

Cross-sectional data were collected from Children’s HealthWatch, an ongoing 5-city network that monitors how economic hardships, including food insecurity, and participation in public assistance programs relate to the health, growth, and development of young children. Between January 1, 2009, and December 31, 2017, caregivers of children<4 years of age were surveyed in emergency departments and primary care clinics in Baltimore, Boston, Little Rock, Minneapolis, and Philadelphia. Children were weighed and measured. Inclusion criteria were English, Spanish, or (Minneapolis only) Somali speaking; state resident where the interview was conducted; and knowledge of the child’s health and development. Critically ill or injured children were excluded. This analysis excluded nonbiological mothers and caregivers with private insurance. Institutional review board approval was obtained before data collection and renewed annually at each site. The total number of interviews representing individual children was 34 739; 433 were excluded because of missing data, 2856 caregivers were not the biological mother, and 3266 had private insurance, with a final sample of 28 184 (Fig 1).

### Measures

The following measures were used:

Demographics: Caregivers provided information on their age, self-identified race and/or ethnicity, country of origin, marital and employment status, highest level of education, and number of household members and children’s age, sex, health insurance, and breastfeeding history.
Food Insecurity: Food security status was assessed by using the 18-item US Household Food Security Survey Module (HFSSM).^23^ The HFSSM includes 10 household-specific and 8 child-specific questions that are used to address food security over the past year. Children were classified into 3 mutually exclusive categories: food secure (FS) if<3 survey questions were endorsed; household food insecure and child food secure (HFI/child secure) if ≥3 non-child-specific questions were endorsed and no more than 1 child-specific question was endorsed; and household food insecure and child food insecure (HFI/CFI) if household food insecure criteria were met and ≥2 of 8 child-specific questions were endorsed.
Growth: Children’s weight and length (height for children> age 2 years) were obtained from medical records. Weight-for-age percentiles and length/height-for-age percentiles were calculated on the basis of World Health Organization and Centers for Disease Control and Prevention standards.^24,25^ Because of missing length/height (*n* = 14 339), obesity was defined as weight-age>90th percentile as recommended when length/height was unavailable.^26^ Underweight was defined as weight-age less than fifth percentile and stunting as length/height-age less than fifth percentile.
Health: Caregiver-reported child health was measured with a question from the 2011–2012 National Survey of Children’s Health for children age 0 to 17 years^27^: “In general, would you say your child’s physical health is excellent, good, fair, or poor?” Responses were categorized as excellent/good or fair/poor.
Development: Caregivers reported on their child’s development using the Parents’ Evaluation of Developmental Status, a validated 10-item caregiver-reported screening instrument.^28^ Caregivers reported any concerns (no, yes, or a little) in response to questions about the child’s development in expressive and receptive language, fine and gross motor skills, behavior, social and emotional, self-help, and preschool performance, and to open-ended questions about concerns in global and cognitive development and “other.” Because the Parents’ Evaluation of Developmental Status has been validated with children>3 months of age, caregivers with infants 0 to 3 months of age were not surveyed with this measure. Children with ≥2 concerns were classified as at developmental risk.^29^
Food assistance: Caregivers responded to questions about their participation in food assistance programs, including the Special Supplemental Nutrition Program for Women, Infants, and Children (WIC) and the Supplemental Nutrition Assistance Program (SNAP). Because both programs can reduce food insecurity among families with young children and improve aspects of diet quality,^30,31^ we adjusted for WIC and SNAP participation. We also asked about participation in other programs (Low Income Housing and Energy Assistance Program and Temporary Assistance for Needy Families).

### Statistical Analysis

χ^2^ tests and analysis of variance were used to characterize the sample. We stratified age into 4 categories: 0 to 12, 13 to 24, 25 to 36, and 37 to 48 months. To examine age-related patterns without considering food security status, we used multivariable logistic regression models to determine if obesity, underweight, stunting, health status, and developmental risk varied by age. The models were run initially without covariates and were then run controlling for covariates previously associated with the dependent measures in our published research, including site; maternal age, education, race and/or ethnicity, marital status, and employment; and child age, birth weight, and food assistance participation.^32^ Obesity and underweight models also included maternal BMI.

Multivariable logistic regressions models were used to determine associations between food security group and obesity, underweight, stunting, fair or poor health, and developmental risk. Analyses were stratified by age category. As the reference, the food-secure group was compared with the HFI/child secure and the HFI/CFI groups. We did not adjust for multiple comparisons because our dependent variables were unique, hypothesized, and measured by multiple methods.^33^ Analyses were performed by using 2-sided tests and a significance level of *P<* .05, with SAS software (version 9.3; SAS Institute, Inc, Cary, NC).

## RESULTS

### Sample Characteristics

Characteristics of study participants were stratified by food security status (Table 1). Of the 28 184 caregiver-child dyads, 72.7% were classified as FS, 14% as HFI/child secure, and 13.3% as HFI/CFI. In this ethnically diverse sample (50% non-Hispanic African American, 34% Hispanic, and 14% non-Hispanic white), 25% of mothers were non-US born, 74% completed high school, 27% met criteria for obesity, 41% were employed, 23% reported depressive symptoms, 65% reported SNAP participation, and 76% reported WIC participation. Children’s mean age was 18.5 months (SD 13.3 months), 46% were female, 14% weighed<2500 g at birth, 64% had been breastfed, 16% were obese, 8% were underweight, and 12% were stunted. Mothers reported that 11% were in fair or poor health and 12% at developmental risk. Food security groups differed on rates of fair or poor health and developmental risk but not on rates of obesity, underweight, or stunting.

### Age-Specific Patterns of Obesity, Stunting, Underweight, Health, and Development

In unadjusted analyses (Table 2), rates of all dependent measures (obesity, underweight, stunting, fair or poor health, and developmental risk) differed by age category (*P<* .001), generally increasing for obesity, fair or poor health, and developmental risk and decreasing for underweight and stunting. In multivariable analyses, 0 to 12 months was the reference. The adjusted odds of obesity were higher among older children, with significant results among the 3 oldest age categories (13 to 24 months: adjusted odds ratio [aOR], 1.1; 95% confidence interval [CI]: 1.01–1.21; 25 to 36 months: aOR, 1.71; 95% CI: 1.55–1.88; and 37 to 48 months: aOR, 2.17; 95% CI: 1.97–2.40) (Table 3). The odds of underweight were higher in the 13 to 24 months category (aOR, 1.19; 95% CI: 1.07–1.3) and significantly lower in the 2 oldest categories, 25 to 36 months (aOR, 0.68; 95% CI: 0.59–0.78) and 37 to 48 months (aOR, 0.49; 95% CI: 0.41–0.78). The odds of stunting were significantly lower across the 3 oldest age categories. The odds of fair or poor health and developmental risk were significantly higher for the 3 older age categories, reaching the greatest odds at 37 to 48 months, fair or poor health (aOR, 1.93; 95% CI: 1.34–2.01), and developmental risk (aOR, 3.44; 95% CI: 3.00–3.95).

### Age-Specific Odds of Obesity, Underweight, Stunting, Health, and Development by Food Security Status

In unadjusted analyses (Table 2), rates of obesity did not differ by food security status at 0 to 12 or 13 to 24 months but rates did differ among the 2 older categories at 25 to 36 and 37 to 48 months. Rates of underweight and stunting did not differ by food security status for any age categories. Rates of fair or poor health differed significantly by food security status among all age categories, with the FS group reporting the lowest rate. Rates of developmental risk differed among the 2 youngest categories, with the lowest rate in the FS group.

In adjusted analyses (Table 3), the FS group was the reference for each age category. There was 1 significant finding related to obesity and none related to underweight or stunting. The adjusted odds of obesity were significantly elevated in the 25 to 36 month category among the HFI/child secure group (aOR, 1.24; 95% CI: 1.01–1.52) but not in the HFI/CFI group. The adjusted odds of fair or poor health were elevated in each of the age categories and across both the HFI/child secure group and the HFI/CFI group. The adjusted odds of developmental risk were elevated in the 0 to 12 month category for the HFI/CFI group (aOR, 1.49; 95% CI: 1.11–1.98) and in the 37 to 48 month category for both the HFI/child secure group (aOR, 1.32; 95% CI: 1.02–1.72) and the HFI/CFI group (aOR, 1.44; 95% CI: 1.12–1.85).

## DISCUSSION

This investigation delineates the relation between food insecurity and obesity, underweight, stunting, health, and development associated with age ranges among children<4 years of age. Rates of overall household food insecurity (27.3%) and child food insecurity (13.3%) exceed national rates reported by the US Department of Agriculture for 2017. Rates of low birth weight (14%) exceed national averages,^34^ and rates of stunting at time of survey (12%) exceed international averages among high-income countries,^35^ revealing the vulnerability of the sample. In this young age sample, rates of obesity were higher among older children (age 37 to 48 months), and rates of underweight were lower, adding to the growing body of research revealing that excess weight gain begins early in life.^36^ The increase in excess weight gain among infants and toddlers continues to be a significant concern, especially in low-income households.^36^

With 1 exception, neither household nor child food insecurity was related to obesity in any age category, consistent with authors of studies that did not examine age stratification.^37^ The exception occurred in the 25 to 36 month category: children in the household but not child food insecure group experienced a 24% increase in the odds of obesity when compared with the FS group. Although the finding could have occurred by chance, a possible explanation is that children aged 25 to 36 months are transitioning from infant-friendly foods to conventional household foods, which, in food-insecure households, may be low-cost, low nutrient-dense food.^38^ In addition, this age period is characterized by variability in children’s appetite and weight gain.^25^ Pickiness and hesitancy to try new foods are transient behaviors that peak in this age group and may be associated with excess snack foods.^39,40^ The association with increasing obesity rates among all groups at 37 to 48 months of age is consistent with findings from a 2015 to 2016 national study that >60% of preschoolers aged 24 to 48 months exceed saturated fat guidelines, increasing their risk of obesity.^41^

The overall lack of association between food insecurity and obesity may be partially due to the increasing rates of obesity overall, suggesting that growing up in a low-income environment exposes many young children to obesogenic factors such as lack of access to healthy grocery stores, overabundance of fast food outlets, and increased screen time, regardless of food security status.^42–44^ It is also possible that chronic stress that can cause inflammation and cycles of overconsumption and restriction, due to the cyclic nature of food insecurity along with the timing of receipt of public nutrition benefits, creates metabolic alterations associated with subsequent weight gain.^45^

Stunting and underweight were not related to either household or child food insecurity in any age category. These findings are consistent with previous studies^46^ and suggest that food insecurity experienced in the United States generally does not result in caloric deficits chronic enough to cause stunting even among children in households reporting child food insecurity.

The health risks associated with food insecurity were demonstrated by increased odds of fair or poor health at every age and increased developmental risk at all ages except 25 to 36 months. Possible explanations are that the poor quality of food in food-insecure households increases the risk for micronutrient deficiencies, which can undermine children’s health and cognitive development^47,48^; that children are exposed to the stress and anxiety that families experience in not having a consistent source of food^4^; and that mothers recognize developmental problems among their children particularly as they age.^28^

Although authors of individual studies have found food insecurity to be associated with reduced diet quality and quantity with nutrient deficiencies among young children,^49–51^ authors of a recent systematic review found inconsistent relations between food insecurity and children’s nutritional status across studies, suggesting the possibility of non-nutritional factors.^52^ Mealtimes in food-insecure households have been described as having increased risk for disruptions in planning and structure.^15^ Food insecurity has been negatively associated with mealtime behavior, increasing the prevalence of restrictive and pressuring styles that preclude the establishment of healthy eating practices.^46,53,54^ It is also possible that parents’ stress and anxiety associated with food insecurity characterize parent-child interactions beyond mealtime because parents who are anxious, depressed, or stressed tend to be less responsive,^55^ and infants of mothers with depressive symptoms, including mothers with previous experiences of adversity and violence exposure are at increased risk for developmental problems.^56,57^ In a longitudinal investigation among infants and toddlers, food insecurity was associated with an increased risk of maternal depression, which was linked to children’s poor health.^46^

Several methodologic issues should be considered. The cross-sectional design does not enable causal interpretations, and the urban sample seeking health care for their children is not representative of the national population. In addition, food security, children’s health status, and children’s developmental risk were reported by mothers, increasing the possibility of shared method variance. However, the use of standardized instruments, systematic data collection, and trained interviewers reduces the possibility of bias. The timing of food insecurity is imprecise because the questions in the HFSSM refer to the previous year, suggesting overlap of timing of food insecurity between categories of age groups. Additional poverty factors related to weight gain, such as limited opportunities for safe play, were not fully captured and controlled. Furthermore, the inclusion of households with marginal food insecurity within the FS group (endorsement of 1 or 2 HFSSM items) and the adjustment for participation in food assistance programs may underestimate food insecurity risks.^58^ We did not adjust for multiple comparisons, increasing the possibility of false-positive findings (type I errors). However, for our main finding that obesity in early life is not associated with food insecurity, the concern is with type II errors, and thus not adjusting for multiple comparisons is conservative. Finally, our focus on obesity, stunting, and underweight reflects the growth parameters associated with subsequent health and development risks and not with anthropometric changes within the normal range.

## CONCLUSIONS

In addition to the urgent need for strategies to eliminate food insecurity, future research should be used to investigate the mechanisms that link food insecurity to adverse health and developmental risk among young children. It is possible that young children in food-insecure households are experiencing stress reactivity, as in reports from older children and adolescents.^59,60^ Research is also needed on strategies to protect families with young children who are experiencing economic hardships from food insecurity.

With these study findings, we illustrate that although children in low-income families and communities continue to experience disproportionately high rates of obesity early in life, in most cases, this disparity is not directly associated with food insecurity. On the other hand, rates of household and child food insecurity are associated with adverse health and developmental conditions among young children. Although some young children in homes experiencing household food insecurity may be shielded from the lack of food availability through caregivers’ positive coping strategies and nutrition assistance programs,^1,61,62^ patterns of detrimental health and development outcomes persist. These findings reveal the importance of considering nonnutritional factors, including caregiver stress and history of adversity, depression, and anxiety, which often co-occur with food insecurity.^4,46,55,63^ Screening questions (Hunger Vital Sign) rather than anthropometric criteria should be used to identify food insecurity, as recommended by the American Academy of Pediatrics.^64^ Using such screening questions can help health professionals refer caregivers to nutrition assistance programs and other supports that can help families cope with economic hardships and their associated stressors.

## Figures and Tables

**TABLE 1:** Characteristics of Sample by Food Security Status (January 1, 2009, to December 31, 2017)

Variable	Overall Children	FS	HFI/Child Secure	HFI/CFI	*P*^a^
Total, *N* (%)	28 184 (100)	20 487 (72.7)	3954 (14.0)	3743 (13.3)	—
Site, *n* (%)					<.0001
Baltimore	5073 (18.0)	3965 (19.4)	642 (16.2)	466 (12.4)	—
Boston	5648 (20.0)	3682 (18.0)	948 (24.0)	1018 (27.2)	—
Little Rock	6114 (21.7)	4629 (22.6)	774 (19.6)	711 (19.0)	—
Minneapolis	4814 (17.1)	2983 (14.6)	838 (21.2)	993 (26.5)	—
Philadelphia	6535 (23.2)	5228 (25.5)	752 (19.0)	555 (14.8)	—
Mothers’ place of birth, *n* (%)					<.0001
US born	21 051 (74.8)	16 067 (78.5)	2766 (70.1)	2218 (59.3)	—
Immigrant	7099 (25.2)	4395 (21.5)	1181 (29.9)	1523 (40.7)	—
Child sex, *n* (%)					
Female	13 107 (46.5)	9517 (46.5)	1869 (47.3)	1721 (46.0)	.5055
Male	15 077 (53.5)	10970 (53.5)	2085 (52.7)	2022 (54.0)	—
Child age, mo					<.0001
Mean (SD)	18.5 (13.3)	18.1 (13.2)	18.9 (13.6)	20.5 (13.5)	—
Median (25th, 75th %)	16.4 (7, 28)	15.9 (7, 28)	16.6 (7, 30)	19.2 (9, 31)	—
Mother ethnicity, *n* (%)					<.0001
Hispanic	9345 (33.5)	6204 (30.6)	1511 (38.5)	1630 (44.1)	—
African American non-Hispanic	13 826 (49.5)	10 582 (52.1)	1744 (44.5)	1500 (40.6)	—
White non-Hispanic	3799 (13.6)	2842 (14.0)	525 (13.4)	432 (11.7)	—
Other	950 (3.4)	673 (3.3)	140 (3.6)	137 (3.7)	—
Married or partnered, *n* (%)					<.0001
No	18 801 (66.8)	13 811 (67.5)	2684 (67.9)	2306 (61.7)	—
Yes	9344 (33.2)	6644 (32.5)	1268 (32.1)	1432 (38.3)	—
Maternal education, *n* (%)					<.0001
Never, elementary, or some high school	7420 (26.4)	4978 (24.3)	1206 (30.5)	1236 (33.1)	—
High school	11 131 (39.6)	8306 (40.6)	1473 (37.3)	1352 (36.3)	—
Technical school, college, master’s degree	9582 (34.1)	7171 (35.1)	1270 (32.2)	1141 (30.6)	—
Mothers					<.0001
*N*	28 080	20 415	3945	3720	—
Age, mean (SD)	27.0 (5.9)	26.5 (5.8)	27.5 (6.2)	28.8 (6.2)	—
Age, median (25th, 75th %)	26.0 (22, 31)	25.0 (22, 30)	26.0 (23, 32)	28.0 (24, 33)	—
Maternal BMI					<.0001
*N*	22 351	16 486	3153	2712	—
Mean (SD)	27.5 (5.3)	27.3 (5.3)	27.9 (5.3)	27.9 (5.3)	—
Median (25th, 75th %)	27.0 (23, 31)	26.6 (23, 31)	27.5 (24, 32)	27.8 (24, 32)	—
Maternal BMI category, *n* (%)					<.0001
Underweight (<18.5)	514 (2.3)	400 (2.5)	60 (1.9)	54 (2.0)	—
Healthy wt (18.5–24.9)	7376 (33.4)	5628 (34.5)	942 (30.2)	806 (30.0)	—
Overweight (25–29.9)	7067 (32.0)	5225 (32.0)	981 (31.5)	861 (32.0)	—
Obesity (≥30)	7157 (32.4)	5059 (31.0)	1132 (36.3)	966 (36.0)	—
Mother employed, *n* (%)					<.0001
No	16 584 (58.9)	11 727 (57.3)	2427 (61.5)	2430 (65.0)	—
Yes	11 557 (41.1)	8729 (42.7)	1522 (38.5)	1306 (35.0)	—
Child breastfed, *n* (%)					<.0001
No	10 146 (36.0)	7861 (38.4)	1272 (32.2)	1013 (27.1)	—
Yes	18 022 (64.0)	12615 (61.6)	2682 (67.8)	2725 (72.9)	—
Depression, *n* (%)					<.0001
No	21 653 (76.9)	16 830 (82.3)	2593 (65.6)	2230 (59.6)	—
Yes	6494 (23.1)	3622 (17.7)	1360 (34.4)	1512 (40.4)	—
SNAP, *n* (%)					.0004
No	9889 (35.3)	7086 (34.9)	1382 (35.2)	1421 (38.2)	—
Yes	18 089 (64.7)	13 242 (65.1)	2548 (64.8)	2299 (61.8)	—
WIC, *n* (%)					.1689
No	6822 (24.4)	5019 (24.6)	930 (23.7)	873 (23.5)	—
Yes	21 194 (75.6)	15 346 (75.4)	3001 (76.3)	2847 (76.5)	—
Current subsidized housing, *n* (%)					.2748
No	22 869 (81.1)	16 669 (81.4)	3192 (80.7)	3008 (80.4)	—
Yes	5315 (18.9)	3818 (18.6)	762 (19.3)	735 (19.6)	—
LIHEAP, *n* (%)					.8363
No	20 685 (83.5)	14 919 (83.4)	2944 (83.6)	2822 (83.8)	—
Yes	4102 (16.5)	2976 (16.6)	579 (16.4)	547 (16.2)	—
TANF, *n* (%)					.0007
No	21 347 (76.1)	15 619 (76.7)	2919 (74.0)	2809 (75.2)	—
Yes	6705 (23.9)	4755 (23.3)	1026 (26.0)	924 (24.8)	—
Low birth wt, *n* (%)					.0716
No	23 903 (86.0)	17 392 (85.9)	3327 (85.1)	3184 (86.9)	—
Yes	3906 (14.0)	2844 (14.1)	583 (14.9)	479 (13.1)	—
Wt-age >90th %, *n* (%)					.6207
No	23 771 (84.3)	17 305 (84.5)	3318 (83.9)	3148 (84.1)	—
Yes	4413 (15.7)	3182 (15.5)	636 (16.1)	595 (15.9)	—
Wt-age <5th %, *n* (%)					.4861
No	25 820 (91.6)	18 778 (91.7)	3604 (91.1)	3438 (91.9)	—
Yes	2364 (8.4)	1709 (8.3)	350 (8.9)	305 (8.1)	—
Height-age <5th %, *n* (%)					.3436
No	13 845 (87.7)	9899 (87.5)	1986 (87.5)	1960 (88.6)	—
Yes	1949 (12.3)	1413 (12.5)	284 (12.5)	252 (11.4)	—
Fair or poor child health, *n* (%)					<.0001
No	25 076 (89.1)	18 496 (90.4)	3421 (86.7)	3159 (84.6)	—
Yes	3071 (10.9)	1967 (9.6)	527 (13.3)	577 (15.4)	—
Developmental risk, *n* (%)					<.0001
No	20 949 (88.5)	15 221 (89.1)	2916 (87.5)	2812 (86.0)	—
Yes	2729 (11.5)	1856 (10.9)	417 (12.5)	456 (14.0)	—

LIHEAP, Low Income Home Energy Assistance Program; TANF, Temporary Assistance for Needy Families; —, not applicable.

a χ^2^ testing was used for categorical variables and analysis of variance was used for continuous variables.

**TABLE 2:** Unadjusted Rates of Obesity (Wt-Age>90th Percentile), Underweight (Wt-Age Less Than Fifth Percentile), Stunted (Height-Age Less Than Fifth Percentile), Fair or Poor Health, Developmental Risk by Age, and Food Security Status

	Age				*P*^a^
	<13 mo	13–24 mo	25–36 mo	37–48 mo	
Obese					
All, *n* (%)	1461 (12.5)	1052 (13.8)	1009 (19.7)	891 (23.9)	<.001^a,b^
Food security status, *n* (%)					
FS	1128 (12.8)	770 (13.9)	714 (19.4)	570 (22.6)	<.001^a,b^
HFI/child secure	176 (10.9)	145 (13.8)	160 (23.1)	155 (26.1)	<.001^a,b^
HFI/CFI	157 (11.9)	137 (13.1)	135 (17.7)	166 (27.3)	<.001^b^
*P* within age category^c^	.06	.77	.03^b^	.02^b^	
Underweight					
All, *n* (%)	1088 (9.3)	787 (10.3)	320 (6.2)	169 (4.5)	<.0001^a,b^
Food security status, *n* (%)					
FS	820 (9.4)	555 (10.0)	217 (5.9)	117 (4.6)	<.001^a,b^
HFI/child secure	148 (9.2)	117 (11.1)	51 (7.4)	34 (5.7)	.001^a,b^
HFI/CFI	120 (9.1)	115 (11.0)	52 (6.8)	18 (3.0)	<.001^a,b^
*P* within age category^c^	.92	.41	.27	.06	
Stunted					
All, *n* (%)	1051 (15.6)	469 (11.6)	241 (8.7)	188 (8.4)	<.0001^a,b^
Food security status, *n* (%)					
FS	778 (15.6)	334 (11.5)	167 (8.6)	134 (9.1)	<.001^a,b^
HFI/child secure	155 (16.7)	73 (12.8)	31 (8.0)	25 (6.5)	<.001^a,b^
HFI/CFI	118 (14.2)	62 (10.6)	43 (10.3)	29 (7.7)	.01^a,b^
*P* within age category^c^	.35^c^	.50^c^	.46	.22	
Fair or poor health					
All, *n* (%)	971 (8.3)	934 (12.3)	659 (12.9)	507 (13.6)	<.001^a,b^
Food security status, *n* (%)					
FS	654 (7.5)	595 (10.8)	415 (11.3)	303 (12.0)	<.001^a,b^
HFI/child secure	164 (10.2)	158 (15.1)	100 (14.4)	105 (17.7)	<.001^a,b^
HFI/CFI	153 (11.6)	181 (17.3)	144 (18.9)	99 (16.3)	<.001^a,b^
*P* within age category^c^	<.001^b^	<.001^b^	<.001^b^	<.001^b^	
Developmental risk					
All, *n* (%)	470 (6.5)	815 (10.7)	836 (16.3)	608 (16.3)	<.001^a,b^
Food security status, *n* (%)					
FS	319 (6.0)	563 (10.2)	572 (15.6)	402 (15.9)	<.001^a,b^
HFI/child secure	77 (7.7)	121 (11.5)	123 (17.8)	96 (16.1)	<.001^a,b^
HFI/CFI	74 (8.7)	131 (12.5)	141 (18.5)	110 (18.1)	<.001^a,b^
*P* within age category^c^	.003^b^	.05^b^	.08^b^	.44^b^	

*N* = 23 678.

a Comparisons across age categories.

b Value reached significance.

c Comparisons within age categories within obese, underweight, stunted, fair or poor health, and developmental risk.

**TABLE 3:** aORs and 95% CIs of Obesity (Wt-Age>90th Percentile), Underweight (Wt-Age Less Than Fifth Percentile), Stunted (Height-Age Less Than Fifth Percentile), Fair or Poor Health, and Developmental Risk by Age and Food Security Status

	<13 mo	13–24 mo	25–36 mo	37–48 mo
Obese (all)^a^	1.00	1.11 (1.01–1.21)^b^	1.71 (1.55–1.88)^b^	2.17 (1.97–2.40)^b^
	Reference	.02	<.001	<.001
Food security status^c^				
FS (reference)	1.00	1.00	1.00	1.00
HFI/child secure	0.85 (0.71–1.01)	0.99 (0.82–1.22)	1.24 (1.01–1.52)^b^	1.18 (0.95–1.47)
HFI/CFI	0.93 (0.77–1.12)	0.94 (0.77–1.16)	0.83 (0.67–1.04)	1.17 (0.94–1.44)
Underweight (all)^a^	1.00	1.19 (1.07–1.32)^b^	0.68 (0.59–0.78)^b^	0.49 (0.41 −0.78)^b^
	Reference	.001	<.001	<.001
Food security status^c^				
FS (reference)	1.00	1.00	1.00	1.00
HFI/child secure	0.89 (0.73–1.09)	1.03 (0.83–1.29)	1.37 (0.98–1.90)	1.14 (0.74–1.75)
HFI/CFI	0.98 (0.78–1.24)	1.08 (0.86–1.36)	1.22 (0.87–1.72)	0.58 (0.34–1.00)
Stunted (all)^a^	1.00	0.69 (0.61–0.78)^b^	0.48 (0.41–0.56)^b^	0.50 (0.42–0.60)
	Reference	<.001	<.001	<.001
Food security status^c^				
FS (reference)	1.00	1.00	1.00	1.00
HFI/child secure	0.98 (0.79–1.20)	1.06 (0.79–1.42)	0.92 (0.61–1.41)	0.63 (0.39–1.02)
HFI/CFI	0.95 (0.76–1.20)	0.91 (0.67–1.23)	1.18 (0.80–1.74)	0.82 (0.53–1.27)
Fair or poor health (all)^a^	1.00	1.59 (1.44–1.75)^b^	1.68 (1.51–1.88)^b^	1.93 (1.71 −2.19)^b^
	Reference	<.001	<.001	<.001
Food security status^c^				
FS (reference)	1.00	1.00	1.00	1.00
HFI/child secure	1.42 (1.18–1.71)^b^	1.53 (1.25–1.87)^b^	1.37 (1.07–1.75)^b^	1.73 (1.34–2.23)^b^
HFI/CFI	1.66 (1.36–2.03)^b^	1.91 (1.58–2.32)^b^	1.85 (1.48–2.32)^b^	1.55 (1.20–2.01)^b^
Developmental risk (all)^a^	1.00	1.90 (1.68–2.15)^b^	3.28 (2.88–3.72)^b^	3.44 (3.00–3.95)^b^
	Reference	<.001	<.001	<.001
Food security status^c^				
FS (reference)	1.00	1.00	1.00	1.00
HFI/child secure	1.12 (0.85–1.47)	1.12 (0.90–1.39)	1.23 (0.98–1.54)	1.32 (1.02–1.72)^b^
HFI/CFI	1.49 (1.11–1.98)^b^	1.22 (0.98–1.53)	1.35 (1.08–1.69)^b^	1.44 (1.12–1.85)^b^

Analyses controlled for site; maternal age, education, race and/or ethnicity, marital status, and employment; and child age, birth wt, and food assistance participation. Obesity and underweight models also included maternal BMI. *N* = 23 678.

a Comparisons across age categories with 0–12 as the reference.

b Significant associations.

c Comparisons within age categories with FS as the reference.

## WHAT’S KNOWN ON THIS SUBJECT:

Food insecurity and obesity are significant public health problems. Among children<4 years of age, food insecurity is associated with poor health, hospitalizations, developmental risk, and behavioral problems. Associations with obesity are inconsistent.

## WHAT THIS STUDY ADDS:

In age-stratified analyses among 28 184 multiethnic, young children, food insecurity was associated with fair or poor health and developmental risk, not with obesity. Screening and referrals may help families cope with economic hardships and associated stressors.

## ABBREVIATIONS

aOR: adjusted odds ratio
CI: confidence interval
FS: food secure
HFI/CFI: household food insecure and child food insecure
HFI/child secure: household food insecure and child food secure
HFSSM: Household Food Security Survey Module
SNAP: Supplemental Nutrition Assistance Program
WIC: Special Supplemental Nutrition Program for Women, Infants, and Children
